# Supplementary material for: Characterization of Macrophages and Osteoclasts in the Osteosarcoma Tumor Microenvironment at Diagnosis: New Perspective for Osteosarcoma Treatment?
Source: Cancers (Basel). 2021 Jan 23;13(3):423. doi: 10.3390/cancers13030423 (PMC7866157; doi:10.3390/cancers13030423)
Supplement: Supplementary file 1 [file cancers-13-00423-s001.zip › Table S1.docx]

| **Patients**  **Total excluded Included ZA- ZA+**  N=124 N= 29 N= 95 N= 60 N= 35 |
| --- |
| **Age**  p = 0.1033 p=0.9018  Median 15.7 14.7 16.0 16.0. 15.9  (Range) (5.7: 50.4) (8.7: 20.9) (5.7: 50.4) (5.7: 48.7) (8.9:50.4)  p = 0.0597 p=0.2724  Age < 18y 85 (68.5%) 24 (82.8%) 61 (64.2%). 41 (68.3%) 20 (57.1%)  Age >= 18y 39 (31.5%) 5 (17.2%) 34 (35.8%)  Missing 0 0 0  **Sex** p = 0.1853 p=0.5797  Male 73 (58.9%) 14 (48.3%) 59 (62.1%) 36 (60.0%) 23 (65.7%)  Female 51 (41.1%) 15 (51.7%) 36 (37.9%) 24 (40.0%) 12 (34.3%)  **Limb vs Axial** p = 0.2964 p = 0.7549  Axial 13 (10.5%) 1 (3.4%) 12 (12.6%) 7 (11.7%) 5(14.3%)  Limb 111 (89.5%) 28 (96.6%) 83 (87.4%) 53 (88.3%) 30 (85.7%)  **Conventional osteosarcoma** p = 0.4930 p= 0.6724  Chondroblastic 31 (25.0%) 6 (20.7%) 25 (26.3%) 18 (30.0%) 7 (20.0%)  Osteoblastic 78 (62.9%) 20 (69.0%) 58 (61.1%) 35 (58.3%) 23 (65.7%)  Fibroblastic 9 (7.3%) 3 (10.3%) 6 (6.3%) 3 (5.0%) 3 (8.6%)  Other 6 (4.8%) 0 (0.0%) 6 (6.3%) 4 (6.7%) 2 (5.7%)  **Meta vs non meta** p = 0.1606 p=0.8927  Localized 100 (80.6%) 26 (89.7% 74 (77.9%) 47 (78.3%) 27 (77.1%)  Metastasis 24 (19.4%) 3 (10.3%) 21 (22.1%) 13 (21.7%) 8 (22.9%)  **Chemotherapy** **p = 0.0374**  p=0.2191  API-AI 13 (10.5%) 0 (0.0%) 13 (13.7%) 6 (10.0%) 7 (20.0%)  MTX 111 (89.5%) 29 (100.0%) 82 (86.3%) 54 (90.0%) 28 (80.0%)  **Chemotherapy response** p = 0.4148 p=0.1663  GR 79 (68.1%) 16 (61.5%) 63 (70.0%) 37 (64.9%) 26(78.8%)  PR 37 (31.9%) 10 (38.5%) 27 (30.0%) 20 (35.1%) 7(21.2%)  Missing 8 3 5 |

Suppl. Table 1. Demographic, clinical and histological data of the entire initial OS2006 patient cohort (n=124)
