# Supplementary material for: Characterization of Macrophages and Osteoclasts in the Osteosarcoma Tumor Microenvironment at Diagnosis: New Perspective for Osteosarcoma Treatment?
Source: Cancers (Basel). 2021 Jan 23;13(3):423. doi: 10.3390/cancers13030423 (PMC7866157; doi:10.3390/cancers13030423)
Supplement: Supplementary file 1 [file cancers-13-00423-s001.zip › Table S2.docx]

|  | **Overall Survival Rate** | |  | **Metastatic progression free survival** | |
| --- | --- | --- | --- | --- | --- |
|  | HR_adj_ [95%CI] | *p* |  | HR_adj_ [95%CI] | *p* |
| **CD8** | 1.5 [1; 2.2] | .058 |  | 1.6 [1; 2.5] | .055 |
| **CD163** | .9 [.7; 1] | .142 |  | .9 [.8; 1] | .085 |
| **CD68** | 1 [.8; 1.2] | .676 |  | 1 [.8; 1.1] | .521 |
| **CSF-1R** | 1 [.8; 1.2] | .776 |  | 1 [.9; 1.2] | .509 |
| **TRAP** | 1 [1; 1.1] | .773 |  | 1 [1; 1.1] | .319 |

Supplementary Table 2. Multivariable biomarker analysis adjusted for prognostic clinical factors. No significant correlation is observed between biomarkers analyzed and overall survival and metastatic progression free survival. (CD8, CD163, CD68: determined by immunohistochemistry; CSF-1R and TRAP: determined in serum.)
